# Submandibular lymph node metastasis of occult thyroid carcinoma first suspected to be a salivary gland tumor: a case report

**DOI:** 10.1186/s13256-021-02901-7

**Published:** 2021-07-23

**Authors:** Hiroyuki Kaneko, Mami Deguchi, Hisashi Yano

**Affiliations:** Department of Oral and Maxillofacial Surgery, Tokyo Women’s Medical University, Medical Center East, 2-1-10, Nishiogu, Arakawa-ku, Tokyo, 116-8567 Japan

**Keywords:** Occult thyroid carcinoma, Papillary thyroid carcinoma, Submandibular lymph node, Neck masses

## Abstract

**Background:**

When diagnosing and treating neck masses, various diseases need to be considered, including benign or malignant tumors, lymph node-related diseases, and cysts. Thus, there may be cases in which making a definitive diagnosis is difficult on the basis of blood testing and imaging alone.

**Case presentation:**

The patient was an 80-year-old Japanese female who presented with swelling in the right submandibular area. Magnetic resonance imaging and ultrasonography revealed a solid tumor with inhomogeneous content continuous with the submandibular gland. Therefore, the clinical diagnosis was salivary gland tumor. Surgical treatment was performed, and intraoperative frozen-section examination demonstrated submandibular lymph node metastasis of thyroid carcinoma. After surgical treatment, blood test for thyroid gland function yielded normal results except for increased thyroglobulin levels. Further positron-emission tomography–computed tomography and ultrasonography were performed, in addition to fine-needle aspiration biopsy of the thyroid gland and other tests; however, no other thyroid abnormalities were observed. Fine-needle aspiration biopsy revealed no carcinomatous components. Close observational follow-up has been continued without thyroid gland treatment, and as of approximately 8 years postoperation, no recurrence, metastases, or thyroid carcinoma have developed.

**Conclusion:**

The mass was lymph node metastasis of occult thyroid carcinoma. In general, occult thyroid carcinoma metastasizes to level II–V. To the best of our knowledge, this is the first report of submandibular lymph node metastasis alone of occult thyroid carcinoma.

## Background

When diagnosing and treating neck masses, various diseases need to be considered, including benign or malignant tumors, lymph node-related diseases, and cysts. If a tumor is suspected, it may be a primary tumor, or lymph node metastasis of a malignant tumor from the oropharyngeal region, or from another organ. Malignant tumors in other organs that can metastasize to the cervical lymph nodes include those in the lung, breast, gastrointestinal system, thyroid gland, ovary, uterus, prostate, and nearly all organs [1]. In cases of metastasis to the upper jugular lymph nodes, the oro- and nasopharyngeal regions are often the primary site of cancer. However, neck tumors discovered in close proximity to the salivary gland, salivary gland tumors, and lymph-node-related diseases are suspected in some cases. Among such cases, it may be difficult to make a definitive diagnosis based on blood tests and imaging alone. Moreover, thyroid carcinoma generally metastasizes to cervical lymph nodes of level II–V [2–5].

We present a case of lymph node metastasis from a thyroid carcinoma in which swelling of the submandibular area was the main symptom and imaging revealed the tumor to be in close proximity to the submandibular gland, suggesting a salivary gland tumor. The final diagnosis of papillary thyroid carcinoma (PTC) was pathologically reached using resected materials; however, additional examinations were unable to detect carcinoma in the thyroid gland. We discuss this rare case, the diagnosis and management of occult thyroid carcinoma (OTC), and metastatic sites of OTC.

## Case presentation

An 80-year-old Japanese female noted swelling of the right side of the submandibular region and underwent a detailed examination at a local hospital. Based on ultrasonography, pleomorphic adenoma was strongly suspected. The patient was referred to our department for further detailed examination and treatment.

**Fig. 1:**
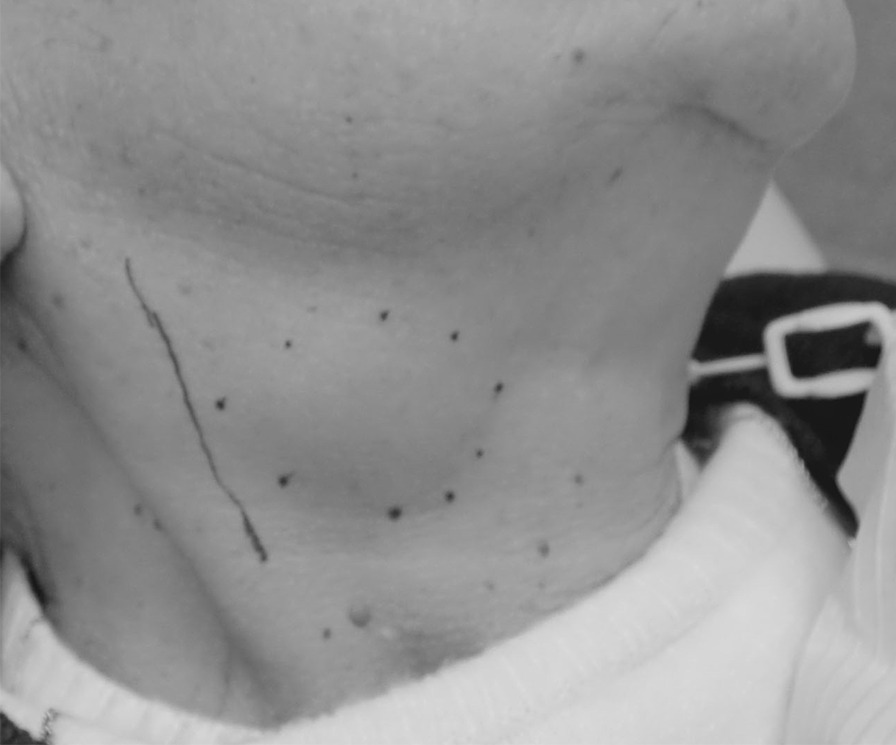
Extraoral findings. An elastic, soft movable tumor measuring 40 × 30 mm was apparent in the right submandibular area

**Fig. 2:**
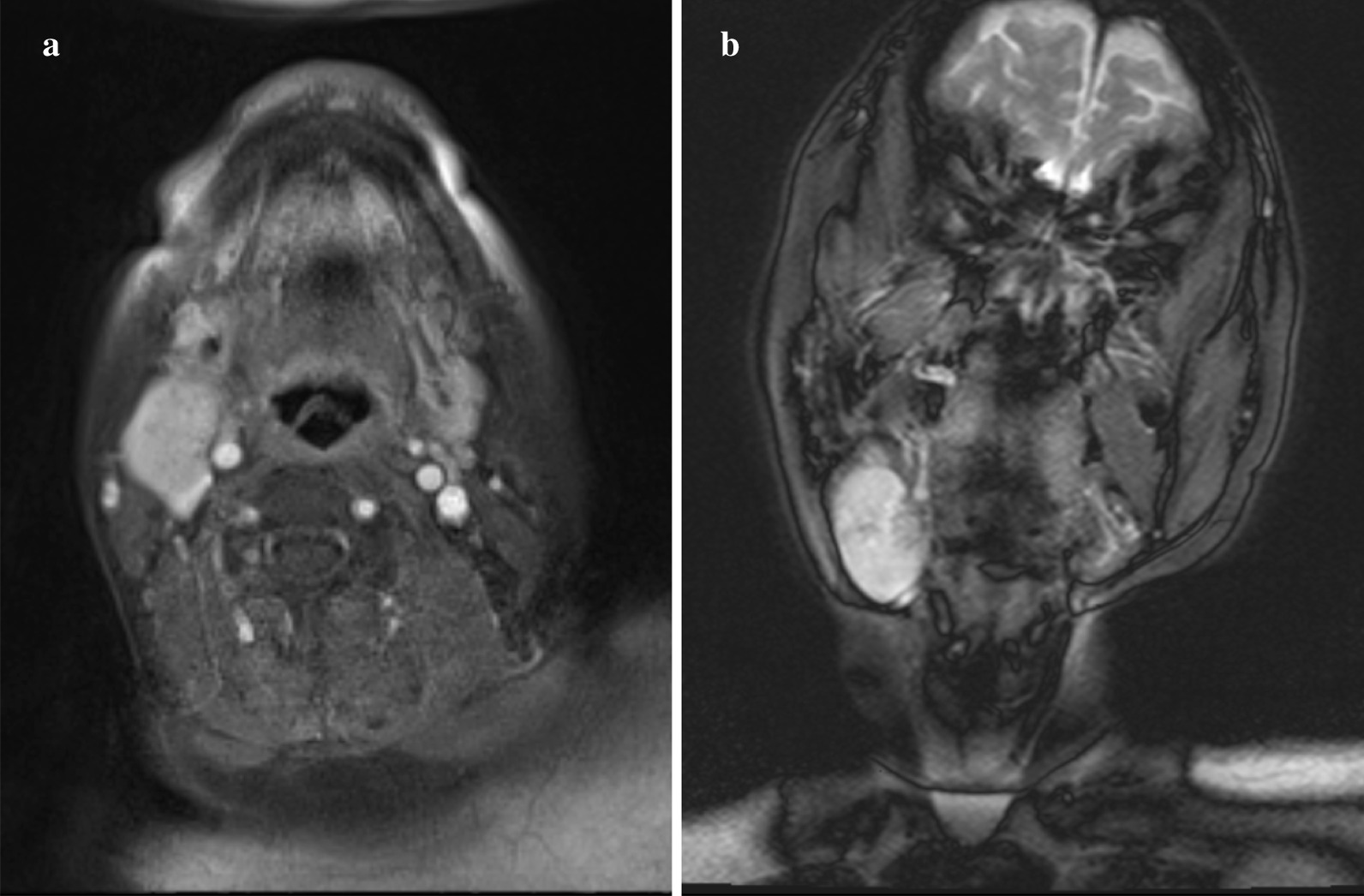
MRI findings. **a** Axial cross section. **b** Coronal section. As shown in **a** and **b**, a tumor measuring 29 × 24 × 40 mm was found exhibiting clear borders between the edges and surrounding tissue. The anterior part of the tumor borders the right submandibular gland, whereas the posterior part was depressing the right internal jugular vein (**a**). In general, the lesion exhibited high-density signal with a low-density area

On initial presentation, an elastic soft and movable mass measuring 40 × 30 mm, with clear borders and healthy skin color, was palpable in the right submandibular area (Fig. 1). No spontaneous pain or pain on pressure was present. Magnetic resonance imaging (MRI) revealed a round mass with relatively clear borders, measuring 29 × 24 × 40 mm, with the anterior edge bordering the right submandibular gland, whereas the posterior deep portion was compressing the right internal jugular vein. The margins were smooth. The mass exhibited a high-signal intensity area that was partially accompanied by a low-intensity signal area in the interior. There was no infiltration into the surrounding tissue, and no other significant lymph node enlargement was observed. Based on these findings, salivary gland tumor was strongly suspected (Fig. 2a, b).

**Fig. 3:**
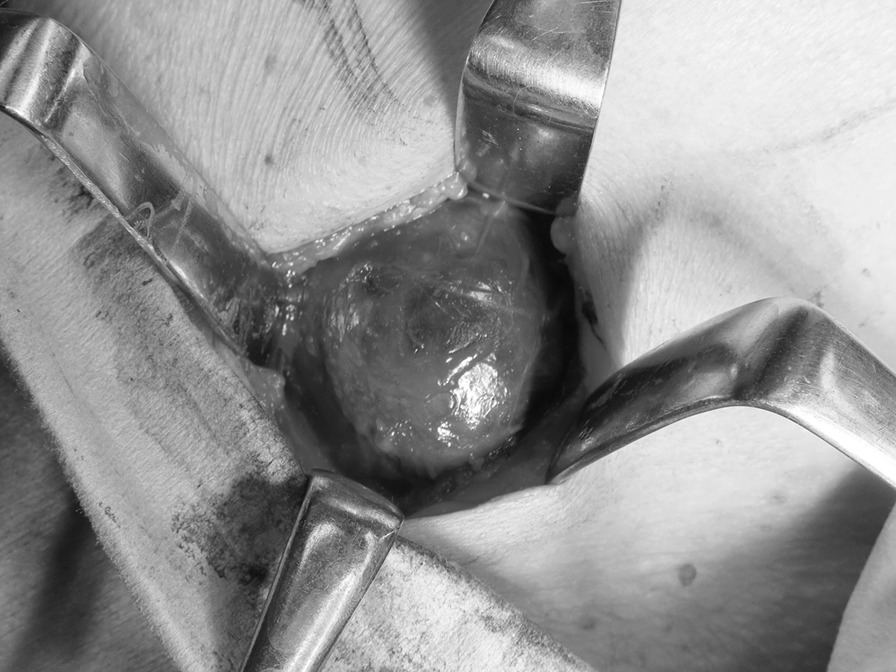
Intraoperative findings. The tumor was round, encapsulated, and dark purple in color

The patient was hospitalized in our department. Two days later, tumor resection was carried out under general anesthesia. The tumor was round, encapsulated, and dark purple in color, and was relatively easily separated from the surrounding tissue. As the tumor was not contiguous with the submandibular gland, part of it was resected, and intraoperative frozen-section biopsy was performed. A large amount of blackish-brown, serous fluid was released from the tumor on resection. Inside the tumor, pale-yellow, papillary structures with a granular appearance were observed projecting toward the interior of the cavity (Fig. 3). Intraoperative biopsy revealed cells that were suspected to have metastasized from the thyroid carcinoma. Based on this diagnosis, the tumor was separated from the surrounding tissue and excised intact. Two lymph nodes that were 12 × 8 mm and 10 × 6 mm in size adjacent to the underside of the tumor were also excised. No adhesion was observed between the tumor and surrounding tissue, and their separation was simple. The wound was washed with saline, and primary closure was performed to complete the surgery.

The final histological diagnosis demonstrated lymph node metastasis of PTC, with a positive reaction for thyroglobulin (Tg) on immunostaining. No metastasis was observed to the other excised lymph nodes. Based on the pathological diagnosis, postoperative examinations were performed, including neck ultrasonography, positron emission tomography (PET)-computed tomography (CT), blood tests [thyroid gland-related serological tumor markers such as Tg, thyroid-stimulating hormone, free triiodothyronine (T3), thyroxine (T4), and carcinoembryonic antigen], upper gastrointestinal digestive tract endoscopy, and plain chest radiography. The serum Tg level was high at 97.2 ng/ml, but no other abnormalities were observed.

**Fig. 4:**
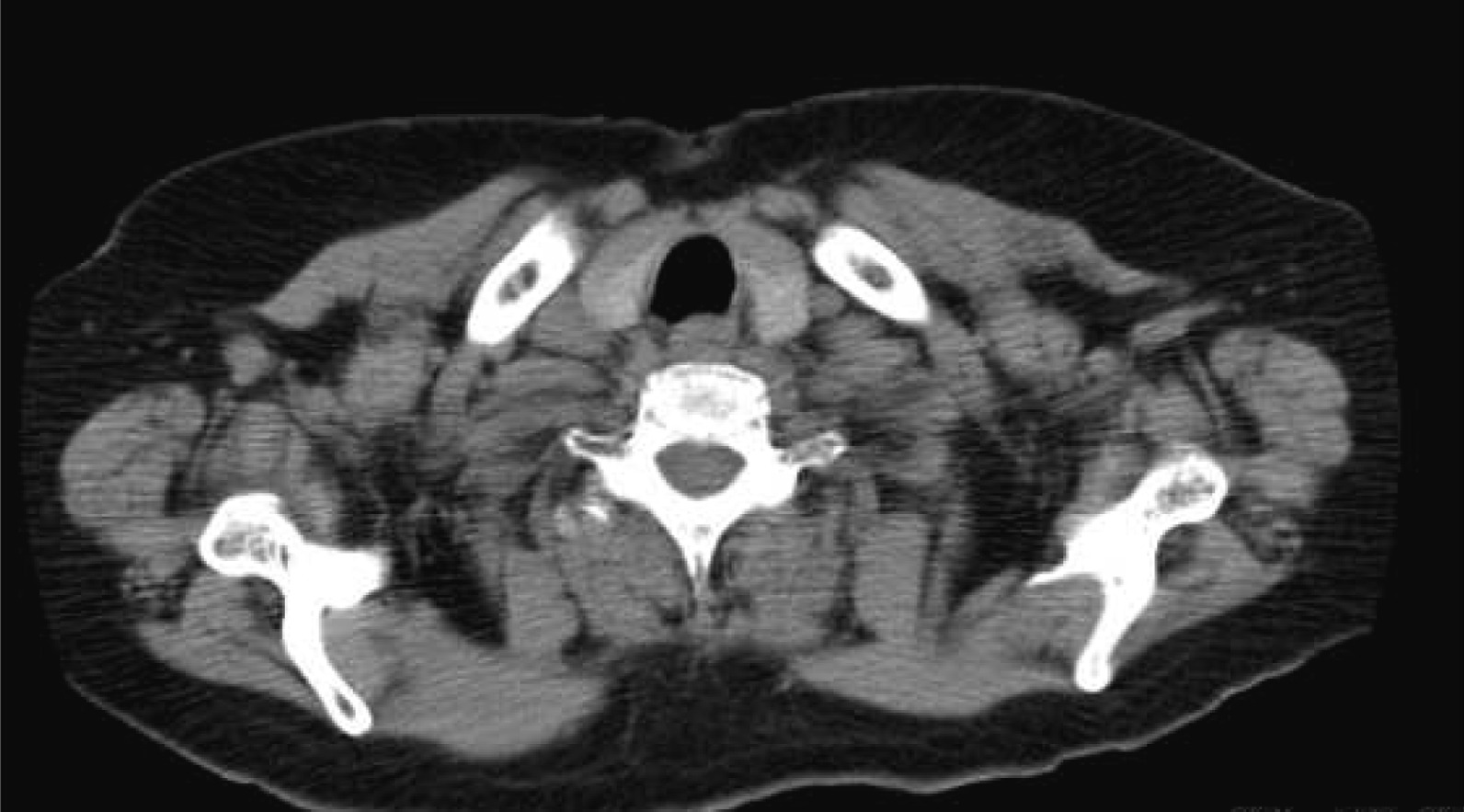
CT findings. The thyroid gland showed normal size, shape, and density

The thyroid gland showed no abnormalities on CT and ultrasonography (Fig. 4). After consultation with the Department of Endocrine Surgery at Tokyo Women’s Medical University Hospital, ultrasonography-guided fine-needle aspiration biopsy was performed twice in 2 months; however, carcinomatous components were not detected in the thyroid gland. In general, cases of cervical lymph node metastasis of occult thyroid carcinoma require total thyroidectomy. In this case, however, considering the slow progression of PTC, the patient’s age, past history of chronic cardiac insufficiency, hypertension, diabetes mellitus, and asthma, and overall physical condition, doctors at Endocrine Surgery could not get informed consent of the patient; therefore, close observational follow-up was selected. As of 8 years after surgery, no recurrence of the neck tumor or change in the size of the thyroid carcinoma has been observed, and the patient is progressing favorably.

### Histopathological findings

**Fig. 5:**
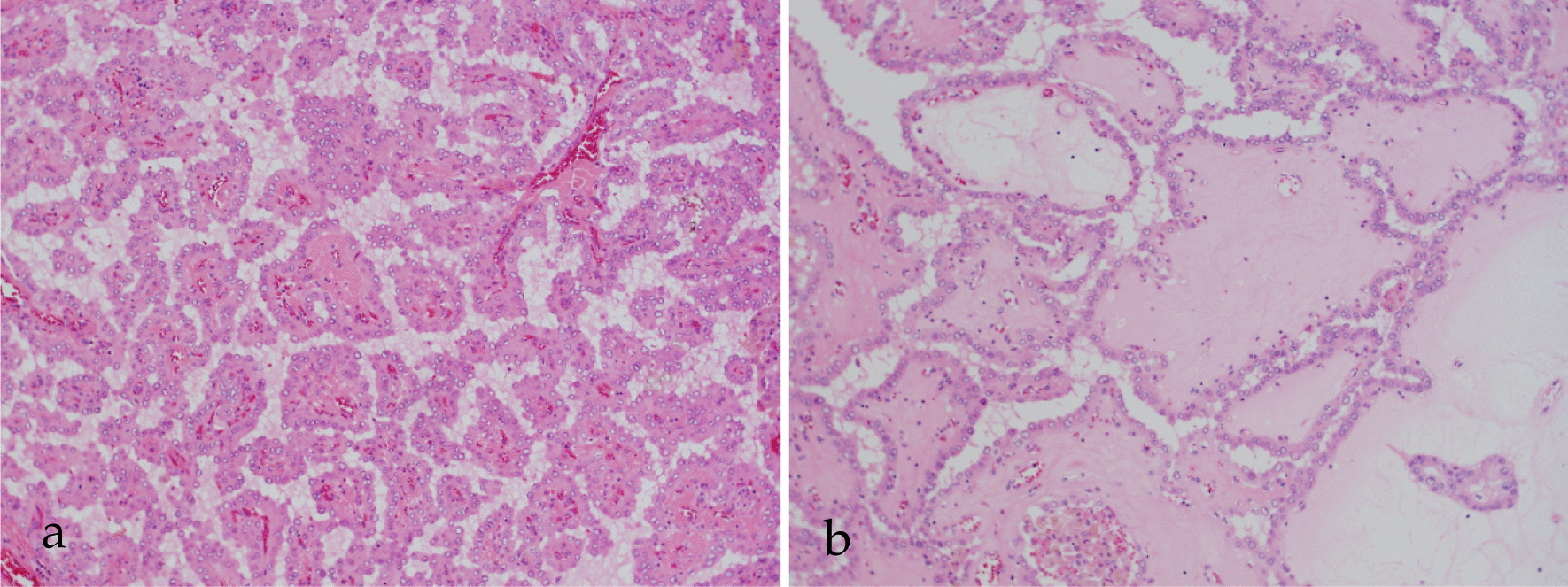
Histopathological examination by hematoxylin and eosin staining. **a** Solid tumor cells have oval nuclei and vacuoles in basophilic, polygonal, or cubic cytoplasm. Tumor cells exhibit papillary proliferation. Slightly basophilic, uniform structures are present in the interstitial tissue and cytoplasm. **b** Some of the tumor cells have a follicular pattern. One or two layers of tumor cells surround the follicles and proliferate in a follicular shape. The follicles are composed of uniform and basophilic material that resembles mucous material

**Fig. 6:**
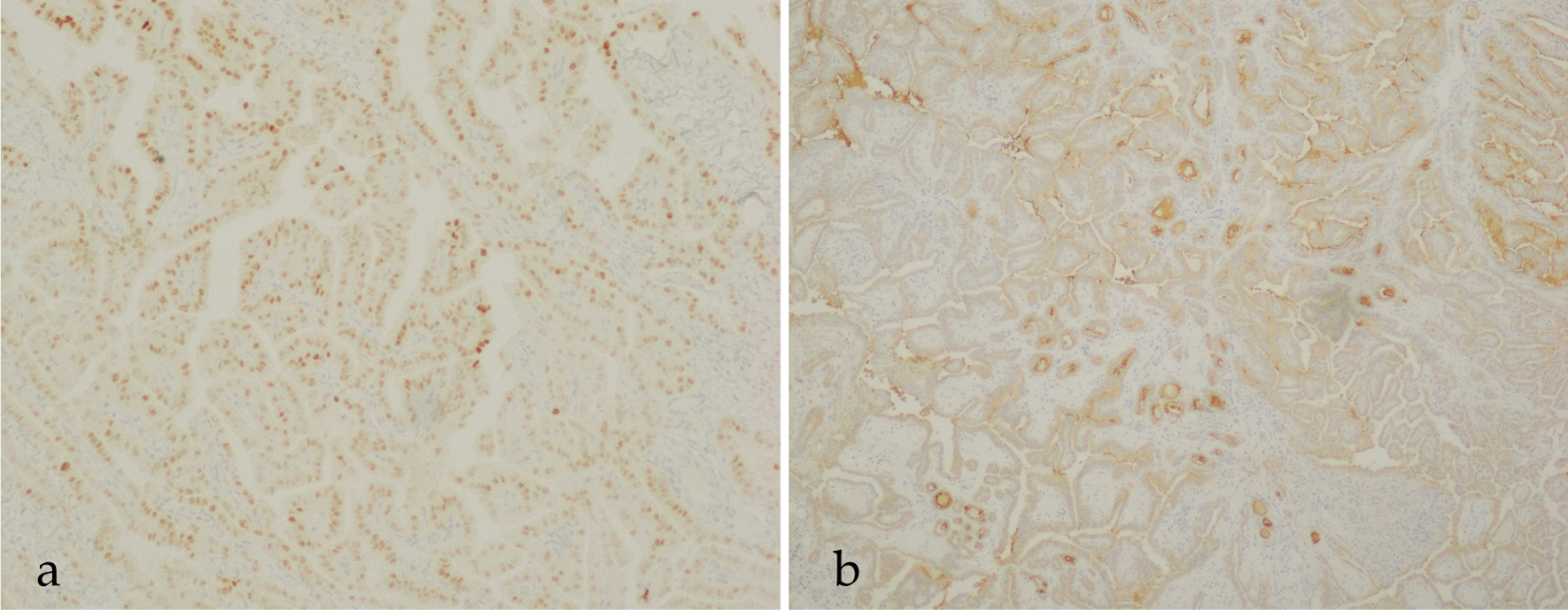
Histopathological examination with immunohistochemical staining. **a** Positive reactions for TTF-1 were detected in the most of nuclei of tumor cells. **b** Positive reactions for Tg were observed in the cytoplasm of tumor cells, and in intrafollicles

Tumor cells had basophilic, polygonal, or cubic cytoplasm, and papillary proliferation. In the cytoplasm, or between cells, uniform structures considered to be slightly basophilic mucin were observed (Fig. 5a). Furthermore, separate from this area, one or two layers of similar tumor cells encompassed the surrounding area and proliferated in a follicular shape (Fig. 5b). Follicles were filled with uniform basophilic structures. Surrounding tumor cells often proliferated into the follicles. There was little mitosis or atypia in the tumor cells. Tumor cell nuclei were positive for thyroid transcription factor-1 (TTF-1), and Tg was positive in the cytoplasm of tumor cells and intrafollicles (Fig. 6a, b). These findings suggested PTC. No metastasis was noted in the other lymph nodes.

## Discussion and conclusions

For neck masses, a therapeutic approach must be selected after conducting the necessary examinations considering tumors, cystic diseases, and lymph node diseases, and identifying the pathological condition. In the submandibular region, in particular, various salivary gland tumors are present, complicating the diagnosis.

In the present case, a diagnosis of benign salivary gland tumor was made on the basis of preoperative image findings, but after intraoperative frozen-section examination, the diagnosis was changed to metastasis from thyroid carcinoma. The final pathological diagnosis was submandibular lymph node metastasis of Tg-positive PTC. As demonstrated in this case, clinical diagnosis can be difficult if it is based only on limited preoperative examinations making it difficult to decide the extent of necessary preoperative examinations. The characteristic pathological findings in this case led to the organ of origin. However, if pathological findings are indefinite, or suggest adenocarcinoma or squamous cell carcinoma, the case should be considered carcinoma of unknown primary origin.

One method for diagnosing neck masses is fine-needle aspiration biopsy (FNAB), which may be effective at identifying tumor components [6, 7]. However, tumor components may not always be retrieved, and if few tumor components are present, a definitive diagnosis may be impossible, necessitating repeat testing. Additionally, when malignant disease present, aspiration must be performed with great caution, owing to the risk of dissemination to the surrounding tissue, especially if the tumor includes fluid. PET-CT is not normally performed when benign salivary gland tumors or cysts are suspected on the basis of preoperative examinations, and performing PET-CT in all cases is difficult. In such cases, even if a benign tumor was suggested as the clinical diagnosis, the condition can be identified at an early stage using intraoperative frozen sections. This is important because, assuming that the diagnosis is lymph node metastasis, the primary lesion can be predicted from the pathological findings in some cases, enabling prompt measures. In general, PET-CT is performed to detect primary carcinoma in cases of lymph nodes metastasis from unknown primary origin after surgery, but primary carcinoma is sometimes undetectable [1, 4, 8–12].

The present case was handled as “OTC.” However, OTC is a general term indicating clinically different situations [13–15]; thus, the incidence of all types of OTC is unknown. Ito *et al*. reported that 17 of 5400 PTC cases (0.3%) were classified as OTC because lymph node metastases were detected by palpation or imaging studies but primary tumors were not identified by ultrasonography. Moreover, there were no apparent carcinoma lesions in the thyroid in five patients, even on pathological examination [13]. In a previous report of metastasis of incidental thyroid carcinoma, lymph nodes metastases were identified in 5 of 1602 neck dissection cases (0.3%) [16]. Therefore, OTC is markedly rare. Keum *et al*. reported that, when overlapping metastases were included, most metastases of PTC were at level IV (75%), followed by level III (69.4%), level II (56.9%), and level V (20.8%), with no metastases at level I. Moreover, 77.8% of PTC metastasized to multiple regional lymph nodes, but metastases at level I alone were again not observed [17]. It is well known that PTC generally metastasizes to cervical lymph nodes of level II–V [2–5]. Therefore, the present case of OTC metastasizing at level I alone is the first to be reported to our knowledge.

Regarding OTC, the subsequent treatment must be carefully decided. In general, apparent or occult PTC in the thyroid gland has a good prognosis, but as its incidence is low, no treatment method has been established. It is unclear whether to perform aggressive treatment for primary and metastatic sites or to only observe and follow up OTC. [14–17]

In the present case, given the age, past history, and general physical condition of the patient, we decided to perform close follow-up. As of 8 years postoperation, no recurrence of the submandibular lymph node, change in the thyroid carcinoma, or lymph node metastasis to other sites has been observed.

When diagnosing submandibular masses, even if a salivary gland tumor is suspected, clinicians must keep in mind that tumors located in a submandibular lymph node may represent malignant metastasis. Therefore, even if the preoperative diagnosis is a benign tumor, intraoperative frozen-section examination is considered important to facilitate early postoperative treatment. Moreover, with OTC, we consider close long-term monitoring of the thyroid gland for metastasis to other sites to be important.

## Data Availability

All data obtained is available within the manuscript.

## Abbreviations

PTC: Papillary thyroid carcinoma
OTC: Occult thyroid carcinoma
MRI: Magnetic resonance imaging
Tg: Thyroglobulin
PET: Positron emission tomography
CT: Computed tomography
TTF-1: Thyroid transcription factor-1
FNAB: Fine-needle aspiration biopsy
